# Evaluating quality management systems for HIV rapid testing services in primary healthcare clinics in rural KwaZulu-Natal, South Africa

**DOI:** 10.1371/journal.pone.0183044

**Published:** 2017-08-22

**Authors:** Ziningi Jaya, Paul K. Drain, Tivani P. Mashamba-Thompson

**Affiliations:** 1 Discipline of Public Health Medicine, School of Nursing and Public Health, University of KwaZulu-Natal, Durban, South Africa; 2 International Clinical Research Center, Department of Global Health, University of Washington, Seattle, Washington, United States of America; 3 Division of Infectious Diseases, Department of Medicine, University of Washington, Seattle, Washington, United States of America; 4 Department of Epidemiology, University of Washington, Seattle, Washington, United States of America; 5 Department of Surgery, Massachusetts General Hospital, Boston, Massachusetts, United States of America; Waseda University, JAPAN

## Abstract

**Introduction:**

Rapid HIV tests have improved access to HIV diagnosis and treatment by providing quick and convenient testing in rural clinics and resource-limited settings. In this study, we evaluated the quality management system for voluntary and provider-initiated point-of-care HIV testing in primary healthcare (PHC) clinics in rural KwaZulu-Natal (KZN), South Africa.

**Material and methods:**

We conducted a quality assessment audit in eleven PHC clinics that offer voluntary HIV testing and counselling in rural KZN, South Africa from August 2015 to October 2016. All the participating clinics were purposively selected from the province-wide survey of diagnostic services. We completed an on-site monitoring checklist, adopted from the WHO guidelines for assuring accuracy and reliability of HIV rapid tests, to assess the quality management system for HIV rapid testing at each clinic. To determine clinic’s compliance to WHO quality standards for HIV rapid testing the following quality measure was used, a 3-point scale (high, moderate and poor). A high score was defined as a percentage rating of 90 to 100%, moderate was defined as a percentage rating of 70 to 90%, and poor was defined as a percentage rating of less than 70%. Clinic audit scores were summarized and compared. We employed Pearson pair wise correlation coefficient to determine correlations between clinics audit scores and clinic and clinics characteristics. Linear regression model was computed to estimate statistical significance of the correlates. Correlations were reported as significant at p ≤0.05.

**Results:**

Nine out of 11 audited rural PHC clinics are located outside 20Km of the nearest town and hospital. Majority (18.2%) of the audited rural PHC clinics reported that HIV rapid test was performed by HIV lay counsellors. Overall, ten clinics were rated moderate, in terms of their compliance to the stipulated WHO guidelines. Audit results showed that rural PHC clinics’ average rating score for compliance to the WHO guidelines ranged between 64.4% (CI: 44%– 84%) and 89.2% (CI: 74%– 100%).Ten out of eleven of the clinics were rated as moderate (70–89%). All clinic have scored highest for the following audit component: equipment; process control and specimen management; and facility ad safety, with 100%. Clinics obtained the lowest scores for the assessment audit component followed by process improvement and organisation, with 40.9% (CI: 15.7–66.1%), 45.5% (CI: 10.4–80.5%) and 56.8% (CI: 31.8 81.8%), respectively. A statistically significant correlation was observed between the following: category of staff performing the HIV rapid tests in the audited clinics and service and satisfactory audit component; weekly average number of patients using the audited PHC clinics and service and satisfactory audit component; number of HIV lay counsellors in the audited clinics and quality control audit component with p<0.05.

**Discussion:**

In the small audit of primary healthcare clinics located within the rural part of KwaZulu-Natal, results revealed an overall moderate rating of the quality management system for rapid HIV testing. Improvements in the organisation, quality control, process improvement and assessment components could enable a higher quality assurance rating for rural HIV testing in KwaZulu-Natal.

## Introduction

In 2013, approximately thirty-five million people were living with HIV globally and of that total, 70% were said to be residing in Sub-Saharan Africa (SSA) [1, 2]. South Africa being home to 25% of the 70% of HIV-infected cases reported in (SSA) [2]. In addition, South Africa has been reported to have the highest HIV infection rate globally [3, 4]. A national survey conducted in South Africa in 2012, revealed a higher HIV prevalence in rural settings than in urban settings [5]. Rural settings in LIMCs are often resource-limited with poor access to quality healthcare services, which in turn has a negative impact on health outcomes [6]. The poor access to healthcare includes poor access to laboratory infrastructure [7]. Rapid diagnostic tests for use at point-of-care have since been developed and utilised in areas where access to quality diagnostic testing is limited.

The main purpose of rapid tests is to provide medical personnel with rapid diagnostic results in order for treatment to be initiated immediately and thus improve health outcomes at a faster rate [8, 9]. As such, implementing rapid HIV testing has been a successful strategy for improving healthcare access and health outcomes in sub-Saharan Africa [10–15], in part because they permit timely initiation of anti-retroviral therapy (ART) and facilitate linkage to care [10–15]. The World Health Organization Consolidated Antiretroviral Drugs Guidelines for Treating and Preventing HIV [16] and the UNAIDS “90:90:90” Strategy [17] recommend the use of innovative rapid HIV diagnostic tests that can be used in various settings, including areas where access to quality laboratory testing is limited. The UNAIDS “90:90:90” strategy aims to ensure that by 2020, 90% of all people living with HIV will know their HIV status, 90% of all people with diagnosed HIV infection will receive sustained antiretroviral therapy and 90% of all people receiving antiretroviral therapy will have viral suppression [17]. Since the introduction of this strategy, the role of HIV rapid tests in diagnosing and facilitating linkage to treatment in various settings cannot be undermined. HIV rapid tests has been associated with decreased mother-to-child transmission of HIV, increased linkage to antiretroviral treatment and care for HIV-infected women [18].

Despite the significant role played by HIV rapid tests in improving health outcomes, the quality of results by POC testing remains questionable since testing is generally conducted by non-laboratory trained personnel in resource-limited settings [19]. Studies highlight an increased risk of misdiagnosis by non-laboratory personnel conducting POC tests, due to their limited knowledge and observation of quality measures for diagnostic testing [20, 21]. According to Gray et al the interpretation of rapid test results is subjective and may impact diagnostic outcomes especially when testing personnel are undertrained [19]. As such, an additional diagnostic test is recommended in order to confirm diagnosis and ensure diagnostic accuracy and reliability [19]. Due to the growing awareness of HIV misdiagnosis, Kosack et al evaluated the most widely used HIV rapid diagnostic tests and the results showed poor performance because sensitivity and specificity of the tests was reduced [22]. When considering the negative emotional and psychological impact that HIV misdiagnosis can have on patients and the financial implications of unwarranted treatment, poor performing HIV rapid tests cannot be accepted [8, 23, 24]. Besides the obvious negative impact of HIV misdiagnosis, rapid test results can also be false negative especially during the early stages of infection [25, 26]. Missed HIV diagnosis often leads to rapid health deterioration of the infected person and prevent early treatment initiation during the early stages of infection [25, 26]. In a world burdened by high HIV prevalence and poor healthcare access in LIMCs, the risk of poor quality testing have a negative effect on linkage to ART and on health outcomes as well as prevent the achievement of the UNAIDS “90:90:90” goals by 2020.

In light of the role of HIV rapid tests to improve access to HIV care and management by providing quick and convenient testing in rural and resource-limited settings [27, 28], it is therefore important to maintain the accuracy and reliability of rapid HIV test results to ensure sustainability of quality service delivery. The World Health Organization (WHO) stipulates quality-**A**ffordable, **S**ensitive, **S**pecific, **U**ser-friendly, **R**apid and robust, **E**quipment-free, **D**eliverable (ASSURED) criteria as a benchmark for point-of-care (POC) tests that are ideal for healthcare facilities in rural and resource-limited settings [29, 30]. Similarly according to the WHO and the Department of Health (DoH) in South Africa, the wide use of rapid HIV tests by various HIV prevention programmes requires accuracy and reliability of diagnostic results in order to be efficient [31, 32].

Preliminary results from our prior survey demonstrated that HIV rapid testing was universally available in rural PHC clinics in KwaZulu-Natal (KZN) [33]. When considering the HIV prevalence statistics previously mentioned, ensuring sustainability and reliability of rapid HIV tests through regular evaluation against set standards is crucial in order to reveal the quality levels of HIV testing services [34]. Therefore since DoH South Africa employs guidelines similar to WHO guidelines for assuring accuracy and reliability of HIV rapid testing for rapid HIV testing in South Africa [32], in this study we aimed to assess the quality management system for HIV rapid testing services in rural PHC clinics in KZN South Africa, against these WHO guidelines. Study outcomes will be useful to policymakers who are responsible for implementing POC diagnostic services in PHC clinics. Results will be shared with the DoH South Africa with the hope that all strengths and shortfalls revealed by the research will be addressed accordingly.

## Material and methods

### Sampling strategy

This is a cross-sectional study, which involved an audit of rural PHC clinics in rural KZN. This study is nested within a large study, which was aimed at revealing barriers and challenges related to implementation of POC diagnostics in rural PHC clinics in KZN [33]. The large started included a survey of 100 clinics aimed at determining the accessibility, availability and usage of POC diagnostic tests in PHC clinics in rural KZN. Multistage sampling was conducted in this study. The initial sampling stage involved proportional stratified sample of 100 clinics from all 11 districts in KZN to ensure generalizability of the survey results. The results of the survey has shown clinics with high availability and usage of POC tests among the 11 districts. HIV rapid tests were shown to be the most universally available and used test in the participating clinics. In order to determine the reliability of the POC diagnostic services in PHC clinics in rural KZN PHC clinics. Therefore, among the 100 participating clinics, one clinic with the highest availability and usage of POC tests per district was included in this study. Clinics with low POC diagnostics availability and usage were excluded in this study (S1 Table).

### Assessment process

For this assessment, we conducted an audit on the sampled clinics from October 2015 to August 2016. The audit team consisted of the study primary investigator (PI), and a trained research assistant, HIV lay counsellors and clinic managers. To ensure efficiency of the audit the audit, HIV lay counsellors and clinic managers were informed about the purpose of the audit and the procedures that will be followed before conducting. The WHO guidelines highlight how the success of POC diagnostics is greatly driven by personnel, quality control and assessment components [31]. The audit tool consists of questions that aim to assess the personnel, quality control and assessment components (S2 Table).

### Assessment tools

For the purpose of this study, we extracted demographic data for each of the participating rural PHC clinics from the survey data set. This extracted data includes the following: the number of lay HIV counsellors per clinic; number of nurses per clinic; average weekly PHC patient census; average weekly number of HIV RT patients; distance to the nearest hospital; and distance to the nearest town from the survey results (S1 Table). The selected clinics were audited against the on-site monitoring checklist adopted from the WHO guidelines for assuring accuracy and reliability of HIV rapid tests [31] (S2 Table).

### Scoring guide

The audit included a series of questions on the following components of the quality management system: organisation; personnel; documents and records; purchasing and inventory; equipment; process control and specimen management; quality control; information management; occurrence management; assessment; process improvement; service and satisfaction; and facilities and safety. The questions consisted of “yes” or “no” responses and were simple and easy for the clinic managers and HIV lay counsellors to understand.

### Data collection

HIV lay counsellors and clinic managers assisted the PI and trained research assistant in conducting the audit. HIV lay counsellors and clinic managers responded to the questions from the aid checklist and helped show the PI and research assistant the different items as per the audit checklist.

### Rating

We summarized the audit scores in order to measure the level of compliance to the WHO guidelines of the clinics in the range of high, moderate, and poor. A high level of quality assurance, defined as a percentage rating of 90 to 100%, refers to strong, reliable, and complete compliance to the stipulated guidelines. A moderate level of quality assurance, defined as a percentage rating of 70 to 89%, refers to adequate but less thorough compliance to the WHO guidelines, with areas of improvement identified. A poor level of quality assurance, defined as a percentage rating of less than 70%, refers to less than satisfactory compliance of the clinics, and overall implementation of the quality management system is not up to the standards stipulated in the WHO guidelines. The percentage rating is calculated as follows:

- One point was allocated to each attribute and question of each component. A point was only allocated when all the requirements for the particular component are fulfilled. If requirements are not fulfilled, then no point was allocated. The total number of points were then converted into a percentages.
- In order to obtain the overall percentage rating, a sum of the scores for each component was obtained to provide the overall percentage rating.

### Statistical analysis

All data were collected manually at site and entered into an excel file, cleaned, validated before it was imported into Stata for analysis. All statistical analysis was conducted using Stata (version 13). Frequencies and 95% confidence intervals (CI) were estimated for all 11 clinic audited clinics using the t-test. We also calculated the interquartile ranges for audit scores to describe the middle 50% of values when ordered from lowest to highest. The relationship between variables were estimated, using the Pearson pair wise correlation coefficient and correlations were reported as significant at p ≤0.05. Linear regression was also calculated to model the relationship between the correlates, the effect size reported using R^2^ and statistical significance at p ≤0.05.

### Ethics statement

Permissions to conduct the audit in the 11 participating PHC clinics were obtained from all 11 KZN district health Managers. Ethical approval for the main study was received from the KZN DoH’s Ethics Committee (HRKM 40/15). Ethical approval for the current study was received from the University of KwaZulu-Natal (UKZN) Biomedical Research Ethics Committee (BE309/16). No informed consents were required for this study.

## Results and discussion

### Characteristics of audited rural PHC clinics in KZN

Eleven rural PHC clinics from KZN province in South Africa were audited from October 2015 to August 2016. Nine out of 11 audited rural PHC clinics are located outside 10 Km of the nearest hospital. All audited clinics are located outside 10Km of the nearest town. uMkhanyakude district clinic was shown to be have the closest proximity (1.1Km) to a hospital, followed by Ugu district clinic (4.1Km). Ugu district clinic was also shown to be at closest proximity to the nearest town (18Km) and Harry Gwala was shown to be the most remote clinic, located 129km from the nearest town. Of the 11 audited PHC clinics sampled from rural KZN, two clinics (Amajuba district and eThekwini district clinic) out of 11 audited PHC clinics reported that HIV rapid test was performed by professional nurses. The highest number of HIV lay counsellors (3) was reported in Ugu district clinic and the lowest number (0) was reported in eThekwini district. uThungulu district clinic was shown to have the highest weekly patient census (11731) and high number of nurses(38). The lowest number of nurses was reported in Amajuba district clinic (5). UThukela district reported the highest number (664) of patients who use the HIV testing services per week and uMgungundlovu reported the lowest number 4 of patients. Summary of the PHC clinic description is shown in Table 1.

**Table 1:** Summary of audit results.

Variable	Mean and standard deviation	Range
**Number of HIV lay counsellors**	1.2±0.9	0–3
**Number of professional nurses**	14.3±9.8	5–38
**Weekly average patient census**	4494.7±3788.5	867–11731
**Weekly census for patients using HIV rapid test**	216.2±171.2	4–664
**Distance from the nearest hospital**	29.3±22.6	11–72.3
**Distance from the nearest town**	42.8±33.2	18–129

### Audit score for the audited rural PHC clinics in KZN

**Fig 1:**
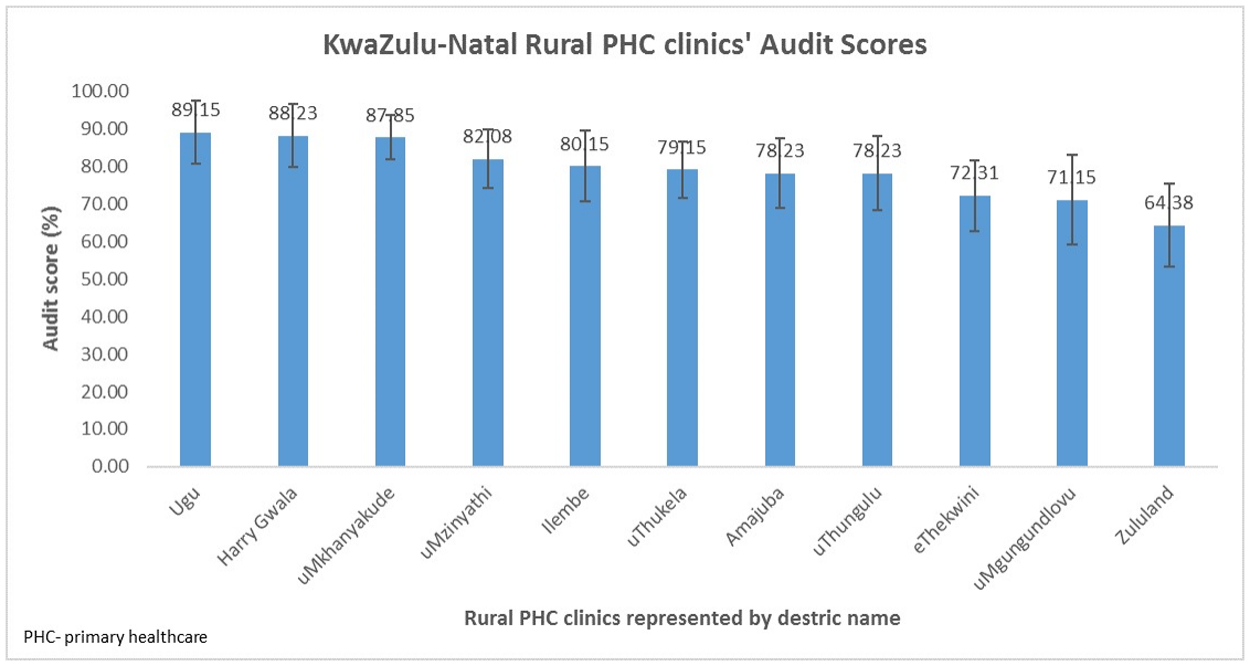
Estimated average audit scores for rural PHC clinics in KwaZulu-Natal audit.

Audit results showed that rural PHC clinics’ average rating score for compliance to the WHO guidelines ranged between 64.4% (CI: 42.1–86.7%) and 89.2% (CI: 72.3%– 100.0%) Table 2 (Fig 1). Based on the rating scale that was applied for this research, ten out of eleven of the clinics were rated as moderate (70–89%). Ugu district clinic obtained the highest audit scores followed by Harry Gwala and uMkhanyakude district clinic with 89.2% (CI: 72.3%– 100%), 88.2% (CI: 71.5–100.0%) and 87.8% (CI: 75.8–99.9%), respectively. The lowest audit scores were observed in Zululand district clinic followed by uMgungundlovu and eThekwini district clinic with 64.4% (CI: 42.1–86.7%), 71.2 (CI: 47.3–95.0%) and 72.3 (CI:53.5–91.1%), respectively.

**Table 2:** Clinic compliance to WHO HIV rapid testing quality standards.

Audit components	Total Score (attributes)	OVERALL PERCENTAGE SCORING PER DISTRICT CLINIC (%)											Overall % of agreement	Component 95% Confidence Interval (95% CI)	
		Amajuba	eThekwini	Harry Gwala	iLembe	Ugu	uMgungundlovu	uMkhanyakude	uMzinyathi	uThukela	uThungulu	Zululand		Lower limit	Upper limit
**Personnel**	4 4	3 (75%)	3 (75%)	3 (75%)	3 (75%)	3 (75%)	4 (100%)	3 (75%)	3 (75%)	4 (100%)	3 (75%)	3 (75%)	79.5 79	72.8 85.5	86.3 73.5
**Documents and records**	6	4 (67%)	4 (67%)	5 (84%)	4 (67%)	5 (84%)	4 (67%)	4 (67%)	4 (67%)	4 (67%)	5 (84%)	3 (50%)	70.1	63.2	77.0
**Purchasing and inventory**	7 7	7 (100%)	6 (88%)	6 (88%)	7 (100%)	7 (100%)	7 (100%)	7 (100%)	7 (100%)	6 (88%)	7 (100%)	6 (88%)	95.6	91.6	99.7
**Equipment**	5	5 (100%)	5 (100%)	5 (100%)	5 (100%)	5 (100%)	5 (100%)	5 (100%)	5 (100%)	5 (100%)	5 (100%)	5 (100%)	100.0 100	100,0 100	100.0 100
**Process control specimen management**	5	5 (100%)	5 (100%)	5 (100%)	5 (100%)	5 (100%)	5 (100%)	5 (100%)	5 (100%)	5 (100%)	5 (100%)	5 (100%)	100.0 100	100.0 100	100.0 100
**Quality control**	6	3 (50%)	3 (50%)	6 (100%)	3 (50%)	6 (100%)	3 (50%)	3 (50%)	3 (50%)	3 (50%)	3 (50%)	3 (50%)	59.1	45.5	72.7
**Information management**	6 6	6 (100%)	5 (83%)	6 (100%)	6 (100%)	6 (100%)	5 (83%)	6 (100%)	6 (100%)	5 (83%)	5 (83%)	5 (83%)	92.3	86.3	98.2
**Occurrence management**	3	3 (100%)	2 (66%)	3 (100%)	3 (100%)	3 (100%)	3 (100%)	3 (100%)	3 (100%)	2 (66%)	3 (100%)	2 (66%)	90.7	80.1	100.0
**Assessment**	8	4 (50%)	0 (0%)	8 (100%)	4 (50%)	8 (100%)	0 (0%)	4 (50%)	4 (50%)	4 (50%)	0 (0%)	0 (0%)	40.9	15.7	66.1
**Process improvement**	1	0 (0%)	1 (100%)	0 (0%)	0 (0%)	0 (0%)	0 (0%)	1 (100%)	1 (100%)	1 (100%)	1 (100%)	0 (0%)	45.5	10.4	80.5
**Service and satisfaction**	7 7	7 (100%)	6 (86%)	7 (100%)	7 (100%)	7 (100%)	7 (100%)	7 (100%)	7 (100%)	7 (100%)	7 (100%)	7 (100%)	98.7 99	95.9 101.2	100.0 96.2
**Facilities and safety**	7 7	7 (100%)	7 (100%)	7 (100%)	7 (100%)	7 (100%)	7 (100%)	7 (100%)	7 (100%)	7 (100%)	7 (100%)	7 (100%)	100.0 100	100.0 100	100.0 100
**OVERALL PERCENTAGE PER CLINIC (95% CI)**		78.2 (59.8–96.7)	72.3 (53.5–91.1)	88.2 (71.5–100.0)	80.2 (61.4 99.0)	89.2 (72.3 100.0)	71.2 (47.3 95.0)	87.9 (75.8–100.0)	82.1 (66.3–97.8)	79.2 (63.9–94.4)	78.2 (58.2–98.2)	64.4 (42.1–86.7)			
**CLINIC RATING**		Moderate	Moderate	Moderate	Moderate	Moderate	Moderate	Moderate	Moderate	Moderate	Moderate	Poor			

**Fig 2:**
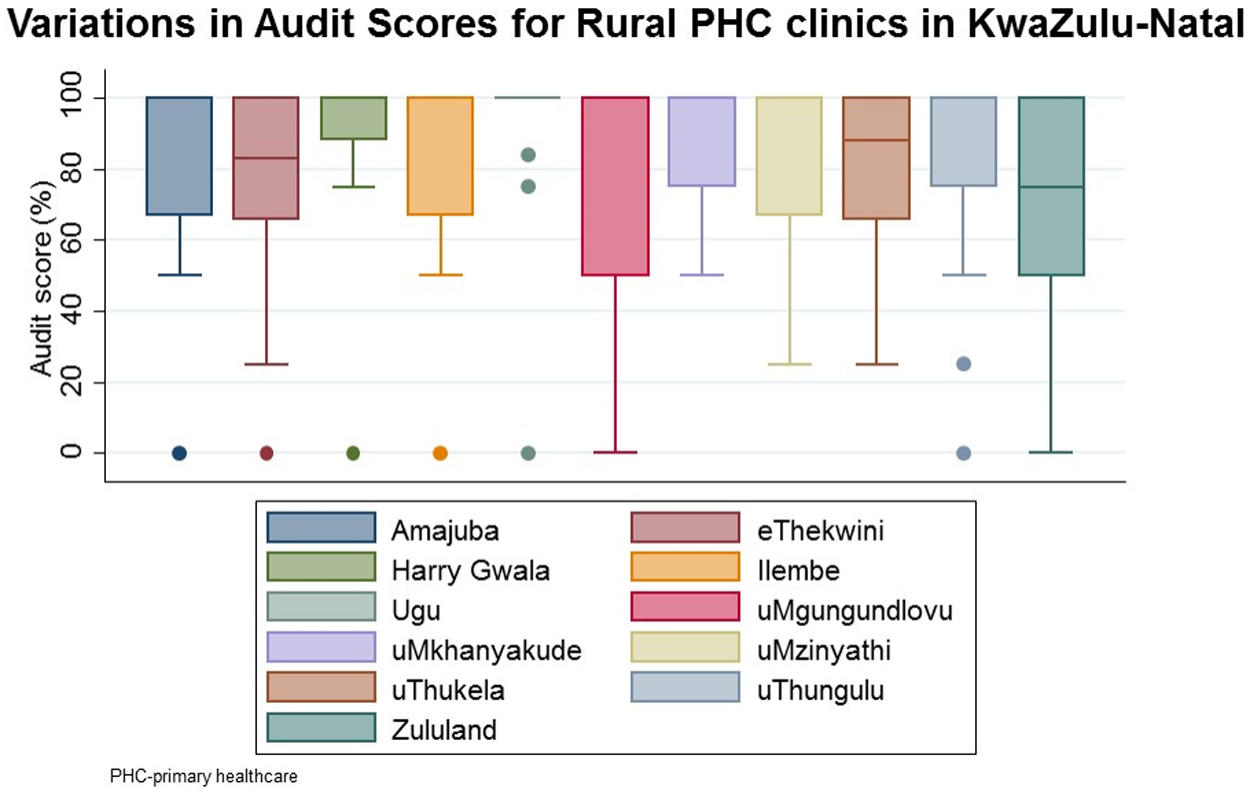
Variations in rural KZN PHC clinics audit scores.

Analysis of variations in audit scores for the 11 audited clinics show that the quality of the HIV rapid test services in these clinics is negatively skewed (Fig 2). uMgungundlovu and Zululand clinics has been shown to have the highest score variation with a 50 interquartile rage (IQR) and Harry Gwala clinic had the lowest score variation with IQR of 12.

### Level of compliance to WHO HIV rapid testing quality standards for audited clinics

**Fig 3:**
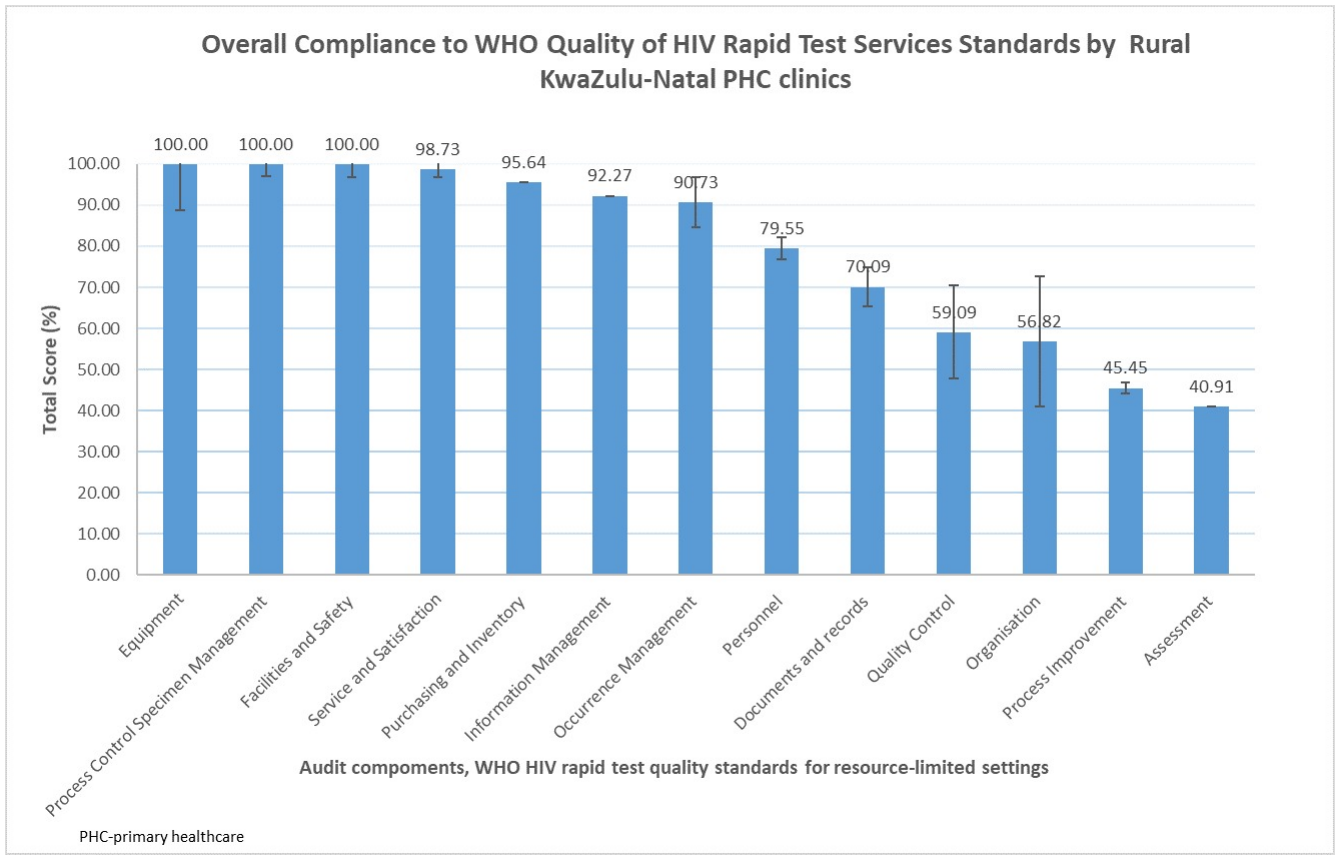
Estimated average audit component scores among the audited rural PHC clinics in KwaZulu-Natal.

The level of compliance to WHO HIV rapid testing quality standards for audited clinics is shown in Fig 3. All clinic have scored highest for the following audit component: equipment; process control and specimen management; and facility ad safety, with 100%. Clinics obtained the lowest scores for the assessment audit component followed by process improvement and organisation, with 40.9% (CI: 15.7–66.1%), 45.5% (CI: 10.4–80.5%) and 56.8% (CI: 31.8 81.8%), respectively.

**Fig 4:**
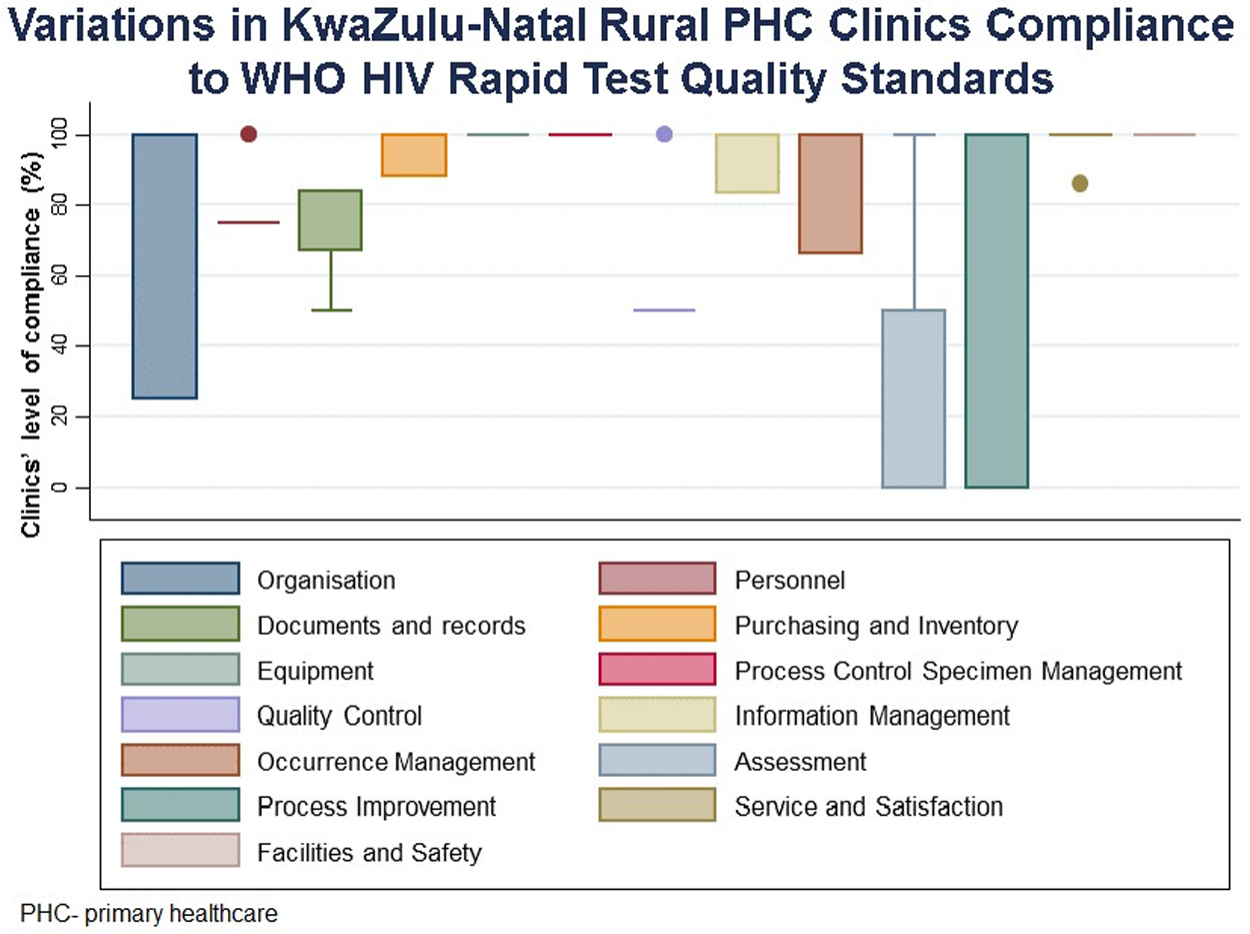
Variations in audit components scores among rural PHC clinics in KwaZulu-Natal.

The combined audit scored for audited clinics show that five (occurrence management, document record, purchase and inventory, information management as well as service and satisfactory) out of 13 audit components are negatively skewed. Process improvement audit component had the highest score variation, followed by organisation and assessment with an IQR of 100, 75 and 50, respectively Fig 4. The lowest score variation was shown in the following audit components: facilities and safety; service and satisfaction; and Process control specimen management with IQR = 0.

#### Organisation

Four of 11 clinics had accessible quality manuals, clinic based quality officers, and clinic managers who were aware of quality systems. As such these clinics demonstrated complete compliance with WHO guidelines for the organisation component, as demonstrated in their audit score rating of 100%. However, six clinics did not have a quality manual and had no quality officers, but the clinic managers had knowledge of quality systems. In addition, only one out of eleven clinics had a quality manual and a clinic manager who was aware of the quality system components, but the clinic had no quality officer. Overall results for seven clinics constituted a 25% compliance with a rating score of 56.8% (CI: 31.8%–81.8%). It was noted that the majority of the clinics indicated that their quality control material is supplied by the South African DoH and only one clinic obtains quality control material from the national laboratory. This clinic is the only one among four that consistently analyse IQC when a new batch of test kits is opened. When considering the above information regarding the supplier of quality control material, the lack of laboratory involvement in the quality aspect of POC diagnostics is clearly revealed. We therefore speculate that the lack of laboratory involvement may be a contributing factor to the overall diminished external quality assurance or proficiency testing EQA/PT programme participation and IQC testing inconsistencies.

#### Personnel

There was consistency at all the clinics regarding staff training and staff conduct. Staff had been trained and were qualified for their positions and all demonstrated professionalism. Despite this, staff at nine of clinics highlighted understaffing as a challenge. Only two clinics expressed that they were coping with their workload. PHC clinics in two districts were the only clinics with complete compliance to the personnel component, with 100%, whereas the rest of the clinics revealed incomplete compliance for this audit component, resulting in a component rating of 79.5% (CI: 72.8%–86.3%).

#### Documents and records

Of the eleven clinics, ten of them did not have a written standard operating procedure (SOP) for HIV rapid testing, but instead followed test kit instructions. Two clinics indicated that they have access to EQA/PT results. However, the other nine clinics indicated they did not have access to EQA/PT results. Ultimately, none of the clinics were fully compliant with the WHO standards regarding this component. None of the clinics completely fulfilled the requirements for documents and records, and presented with an overall 70.1% (CI: 63.2%–77.0%) rating score for this audit component.

#### Purchasing and inventory

There was consistency among all the clinics regarding correct kit storage and use. Four out of eleven audited clinics reported having experienced a shortage of HIV rapid test kits in the past. When this occurred, at three of the clinics, the patients were informed of the shortage and asked to return to the clinic at another time. However, at one clinic the patients were sent to neighbouring healthcare facilities. PHC clinics in six districts demonstrated full compliance to the guidelines for purchasing and inventory, with a 100% rating score, which resulted in a 95.6% (CI:91.6%– 99.7%) component rating score.

#### Equipment

All eleven audited clinics reported that they conduct regular maintenance of the refrigerator by checking the temperatures twice a day. A complete compliance of 100% score, across all the clinics regarding equipment, process control specimen management, and facilities and safety, was observed, thus leading to 100% rating score for all these three components.

#### Process control—Specimen management

At all the clinics, staff observed universal precautions when collecting and handling patient specimens. Five out of eleven audited clinics did not have an SOP for specimen collection, labelling and transport, whereas six clinics did have an SOP. At two clinics, observation of HIV rapid testing by staff could not be performed. Observation of staff at two clinics could not be performed because the clinics were about to close for the day and there were no patients present. However, at nine clinics staff was observed and they followed test kit instructions. A complete compliance of 100% score, across all the clinics regarding equipment, process control specimen management, and facilities and safety, was observed, thus leading to 100% rating score for all these three components.

#### Quality control

The audit revealed that of the eleven participating clinics, a total of nine did not participate in any EQA/PT programme. However, two clinics indicated participation in an EQA/PT programme. There was consistency among all the clinics regarding internal quality control (IQC) material analysis, and all clinics indicated that they do analyse IQC. Four clinics indicated that IQC analysis is performed when a new batch of test kits is opened. One clinic indicated that IQC analysis is performed every Monday. Three clinics indicated less frequent EQA/PT control analysis, once a month to once in three months. Moreover, three clinics did not indicate frequency of IQC controls testing. Seven clinics indicated the DoH as their supplier for the quality control material, but only one clinic indicated obtaining quality control material from the National Laboratory. One clinic indicated that they use patient samples for EQA/PT analysis in association with the local laboratory. The source of quality control material is unknown for two clinics. Only two clinics were fully compliant to quality control requirements with a 100% rating score. These results place the quality control component rating score at 59.1% (CI: 45.5%–72.7%).

#### Information management

All participating clinics consistently maintain patient confidentiality. Five clinics do not use computers to back-up patient results, only handwritten results are kept, whereas six clinics use computers to back-up patient results. Trained data capturers enter the HIV rapid test information into the computer to help ensure accuracy of results and maintain integrity of data at six clinics. To ensure patient confidentiality, clinics have a designated area for HIV rapid testing and a designated file patients’ HIV RT testing records including test results. The HIV rapid test file is kept in a locked cupboard within the clinics. All eleven clinics meet the programme reporting requirements. In terms of information management, only seven clinics were fully compliant to the requirements, resulting in a rating score of 92.3% (CI: 86.3%–98.2%).

#### Occurrence management

All eleven clinics did not have a policy for reporting and recording results. However, nine clinics indicated that they follow the South African DoH, guidelines for reporting and recording errors. No copies of the DoH guidelines was found on site. All participating clinics indicated that no errors had occurred thus far. Seven clinics out of the 11 audited PHC clinics demonstrated full compliance to occurrence management requirements. As a result, this component achieved a rating score of 90.7% (CI: 80.1%–100%).

#### Assessment

Nine clinics out of the eleven audited clinics indicated that they did not participate in any EQA/PT programme, and only two indicated participation. Turnaround time for re-tested samples and corrective action, were not applicable to the nine clinics since they do not participate in any EQA/PT programme. The two clinics indicated that re-testing of samples has not occurred, and corrective action would be conducted if required. Four clinics had not been assessed within the past six months, whereas seven clinics indicated that an assessment had occurred within the past six months. Two clinics out of 11 audited PHC clinics indicated 100% compliance. There was no compliance at all, 0%, to this component by four PHC clinics. However, partial compliance of 50% was noted at three clinics. Therefore, the rating score for the assessment component was 40.9% (CI: 15.7%–66.1%).

#### Process improvement

The assessment included questions regarding clinics undertaking projects to improve clinic processes. A total of six clinics indicated that no process improvement interventions had been undertaken, whereas five indicated that process improvement interventions had been initiated.

eThekwini, uMkhanyakude, uMzinyathi, uThukela and uThungulu district clinics were the only clinics that demonstrated 100% compliance to the requirements for this component, whereas there was 0% compliance at the other six clinics. As such, this component was rated at 45.5% (CI: 10.4%–80.5%). These clinics reported that they receive regular training programme for healthcare workers involved in HIV rapid testing.

#### Service and satisfaction

Staff at all participating clinics were courteous to the clients and there was an effort to alleviate patient’s fears. There was adequate space in the testing rooms of ten clinics. However, one clinic did not have sufficient space for testing. One out of 11 audited PHC clinics, eThekwini, demonstrated partial compliance to this component, at 86%, and the rest of the components were fully complied with, thus resulting in a rating score of 98.7% (CI:95.9%–100.0.%).

#### Facilities and safety

There is a policy for addressing accidental exposure to infectious material, and all staff are aware of post exposure prophylaxis. A complete compliance of 100% score, across all the audited PHC clinics regarding equipment, process control specimen management, and facilities and safety, was observed, thus leading to 100% rating score for all these three components.

### Relationship between audited PHC clinic characteristics and audit scores

The audit scores and clinic demographic data were compared to determine significant relationships. The results showed that there was a significant positive correlation between the category of staff performing the HIV rapid tests in the audited clinics and service and satisfactory audit component with r = 0.7 and p<0.05. The service and satisfactory audit component was also shown to be significantly correlated with the weekly average number of patients using the audited PHC clinics with r = 0.6 and p<0.05. The number of HIV lay counsellors in the audited clinics have been shown to be significantly correlated with the quality control audit component with r = 0.7 and p<0.05.

Further analysis of the correlates, category of staff performing the tests and service and satisfactory audit component with the linear regression model has shown a statistically significant correlation. The linear regression model established that category of healthcare worker who perform HIV rapid test could significantly predict the service and satisfactory for HIV rapid test services, F (1.9) = 7.3, p = 0.02. The estimated effect size for this correlation is R^2^ = 50%. Linear regression analysis has also established that weekly average number of patients using the audited PHC clinics could also significantly predict that service and satisfactory for HIV rapid test services, F (1.9) = 5.93, p = 0.05. The estimated effect size is R^2^ = 40%, p<0.05. The regression model for number of HIV lay counsellors in the audited clinics and quality control audit component has been shown to be statistically correlated with the highest effect size with R^2^ = 60%, p<0.05.

## Discussion

This study assessed the quality management systems for HIV rapid testing services in rural South Africa, through and audit of 11 rural PHC clinics with high availability and usage of HIV rapid test in rural KwaZulu-Natal province. Audit results revealed that majority of rural KZN PHC clinics were moderately compliance to the WHO HIV rapid testing quality standards [31]. The highest combined compliance score was shown on equipment, process control specimen management and facility safety. Although clinics obtained a moderate score the document standard, they used the manufacturer’s instructions to perform the test. Audited clinics obtained the lowest combined compliance score on the assessment, followed by process improvement, organisation and quality control standard. Lacked access to quality manuals and clinic based quality officers was also observed from the majority of the audited clinics attributing to a poor score for the organisation component. Ugu district PHC clinic scored the highest overall quality score and Zululand district PHC clinic scored the lowest overall score. Our study results show a statistically significant positive correlation between the following: category of staff performing the HIV rapid tests in the audited clinics and service and satisfactory audit component; weekly average number of patients using the audited PHC clinics and service and satisfactory audit component; and number of HIV lay counsellors in the audited clinics and quality control audit component.

To the best of our knowledge this is the first study to assess the quality of implementing HIV QMS in rural PHC clinics with an HIV high pandemic region. The use of WHO HIV rapid test quality standards [31] for the audit has enabled adequate assessment of the services in rural PHC clinics. The study findings has contributed to gaining a better understanding of HIV rapid testing in rural PHC clinics and exposed areas that require attention to improve the level of service being provided to the clients or patients. The 11 rural PHC clinics audited in this study were used as a model for other rural PHC clinics in South Africa. Therefore findings of this study can be generalised to other PHC clinics and for other routine POC testing conducted outside laboratory setting. This study emphasizes the need for quality HIV testing in line with the rapid HIV testing quality improvement initiative (RTQII) [35]. This study is also relevant to the new UNAIDS 90:90:90 target and its findings has been supported by previous research [17]. The study has shown lack of documentation, this finding is in agreement with findings by Lewandrowski et al, 2011 and Dyhdalo et al, 2014 which highlighted that regulatory compliance as a great challenge for POC testing [36, 37]. The results of our audit show inconsistencies in the duration of IQC analysis for every new test kit lot number, these findings are also supported by Lewandrowski et al, 2011 and Dyhdalo et al, 2014 studies, which highlighted that non-laboratory workers conducting POC testing may not fully understand the importance of quality control and documentation and therefore neglect to comply with regulations for POC diagnostics [36, 37]. Our study findings on poor staffing for PHC clinic staff supports findings of a systematic review by Pai et al, 2015, which revealed understaffing as challenge for primary healthcare workers attending to a large number of patients [38]. Poor process improvement was also shown in a majority of the clinics, according to the WHO guidelines for assuring accuracy and reliability of HIV rapid testing, process improvement is part of a continuous programme to identify gaps in the system and develop strategies to close these [31]. Despite efforts by various organisations to improve access and availability of resources, many facilities, including healthcare facilities, located in rural and resource-limited settings continue to have diminished resources as indicated by lack of back-up system for HIV rapid test results. Previous studies support this finding by highlighting how healthcare in these areas is plagued by resource shortages [39–41]. Our study demonstrate a statistical significant correlation between category of staff performing the HIV rapid tests and service and satisfactory audit component in line with the findings of earlier study conducted in KZN, which revealed that reliability of HIV rapid testing is user dependent [42].

Despite the importance of the data generated in this study, it is important to highlight one limitation. Although the findings of this study may also be a reflection of the state of the quality management system of POC diagnostics in general, the audit tool adapted from the WHO was not specifically designed for rural and resource-limited settings, so some of the assessed aspects may not be applicable to rural PHC clinics in South Africa. The results of our audit show that there were audited clinics obtained a high score for the occurrence management component and there were no HIV rapid testing errors reported in all clinics, the absence of a written policy for investigating errors in majority of the clinics could have resulted poor error reporting. Furthermore, the small sample size yielded a wide CI range which indicates that little is known about the true value of the mean and therefore further research with a larger number of sample is required in order to improve precision for estimating the parameter of interest.

### Recommendations

The audit results has shown the following deficiencies in the quality of HIV rapid testing services in audited PHC clinics in rural KZN: lack of access to quality manuals; lack of clinic based quality officers; lack of SOPs for testing; lack of EQA/PT analysis; inconsistency in IQC testing frequency; understaffing; lack of back-up systems for patient results; lack of process improvement interventions; and the rare test kit depletion. Based on these findings we recommend the following:

- Quality manuals should be made available to the clinics and accessible to staff, and other healthcare facilities located in rural and resource-limited settings should adopt this practice.
- All clinics should nominate a quality officer for POC diagnostic services.
- There should be EQA/PT participation and consistent IQC testing for clinics that are not currently participating in an EQA/PT programme for POC diagnostics services.
- An alliance with the national laboratory should be made to facilitate EQA/PT participation
- Supply chain management should be strengthened for POC diagnostics to prevent depletion of test kits at the clinics.
- Process improvement interventions should be initiated to identify and monitor clinic testing processes and procedures.
- Each healthcare facility should have its own SOP for testing, result interpretation and recording of results.
- We recommend the use of dry blood specimen (DTS) EQA/PT for HIV testing quality monitoring in rural PHC clinics. This is cost effective EQA/PT technology recommended for rural and resource-limited settings. Use of this technology could help with monitor HIV testing accuracy for continual quality improvement in rural PHC clinics. In addition, this technology could address logistic challenges, coverage, registration and subscription cost associated with traditional EQA/PT system.
- A follow-up assessment/audit of the assessed 11 PHC to measure progress made toward quality improvement in HIV testing in those clinics.

## Conclusion

The results of this study confirm that the quality management system for HIV rapid testing in rural PHC clinics in KZN is moderate. Poor adherence to the WHO HIV rapid testing services standards for quality control, organisation, process improvement and assessment have been demonstrated in the audited clinics. However, it is important to note that these findings may be biased since the audited clinics do have the highest usage of HIV rapid tests and as such the focus on HIV POC testing may be higher compared to the other PHC clinics. HIV rapid testing has been previously highlighted as the most widely used and most impactful POC diagnostic test in the fight against the HIV pandemic. For this reason, complete overall compliance to recommended guidelines would be expected. Based on these findings, it is clear that much effort has been placed on the quality management system of HIV POC testing in rural PHC clinics, however there is much work is still required in order to improve the system. Therefore, we suggest a review of overall implementation strategies for POC diagnostics in these settings.

## Supporting information

S1 Table Characteristics of the 11 participating primary healthcare clinics in rural KwaZulu-Natal and audit dates. (DOCX)

S2 Table Audit tool. (DOCX)
