# Supplementary material for: Evaluating quality management systems for HIV rapid testing services in primary healthcare clinics in rural KwaZulu-Natal, South Africa
Source: PLoS One. 2017 Aug 22;12(8):e0183044. doi: 10.1371/journal.pone.0183044 (PMC5567898; doi:10.1371/journal.pone.0183044)
Supplement: S1 Table — (DOCX) [file pone.0183044.s001.docx]

**S1 Table. Characteristics of the 11 participating primary healthcare clinics in rural KwaZulu-Natal and audit dates.**

| **KZN District** | **Date of audit** | **Number of HIV lay counsellors** | **Number of nurses** | **Average weekly PHC headcount** | **Average weekly number of HIV RT patients** | **Distance to nearest hospital (Km)** | **Distance to nearest town (Km)** |
| --- | --- | --- | --- | --- | --- | --- | --- |
| **Amajuba** | 15 August 2016 | 0 | 5 | 867 | 150 | 28,2 | 30 |
| **eThekwini** | 21 September 2015 | 0 | 19 | 11 379 | 250 | 33 | 44 |
| **Harry Gwala** | 17 August 2016 | 2 | 14 | 2 558 | 200 | 72,3 | 129 |
| **Ilembe** | 19 July 2016 | 1 | 15 | 2 495 | 250 | 39 | 45 |
| **Ugu** | 16 May 2016 | 3 | 10 | 4 862 | 150 | 1,4 | 18 |
| **uMgungundlovu** | 08 October 2015 | 1 | 24 | 5 855 | 4 | 61 | 76 |
| **uMkhanyakude** | 16 July 2016 | 1 | 9 | 3 828 | 150 | 1,1 | 21 |
| **Umzinyathi** | 17 July 2016 | 2 | 6 | 2 023 | 60 | 8,6 | 36,4 |
| **uThukela** | 18 December 2015 | 1 | 10 | 2 657 | 664 | 21,8 | 18,8 |
| **uThungulu** | 14 December 2015 | 1 | 38 | 11 731 | 300 | 20,6 | 20 |
| **Zululand** | 15 December 2015 | 1 | 7 | 1 187 | 200 | 34,8 | 33,3 |
