# Supplementary figures and images for: Evaluating quality management systems for HIV rapid testing services in primary healthcare clinics in rural KwaZulu-Natal, South Africa
Source: PLoS One. 2017 Aug 22;12(8):e0183044. doi: 10.1371/journal.pone.0183044 (PMC5567898; doi:10.1371/journal.pone.0183044)

**S2 Table: Audit Tool**


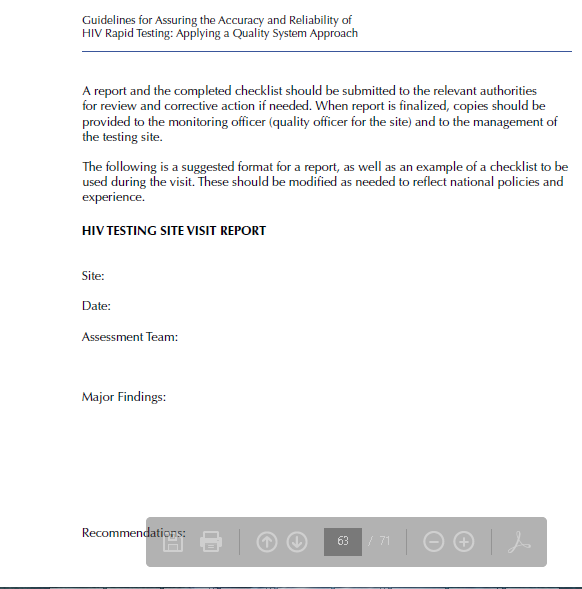


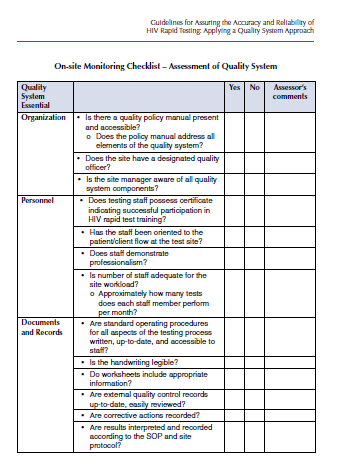


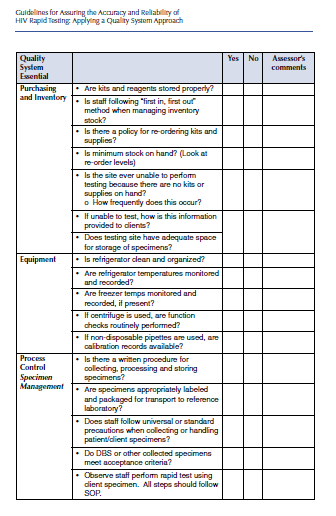


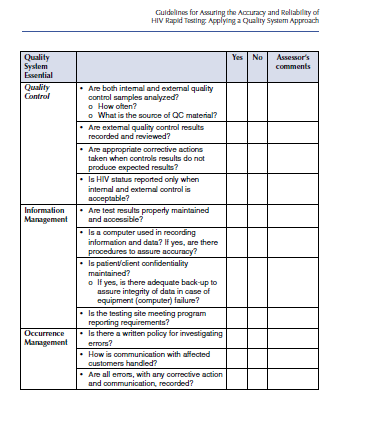


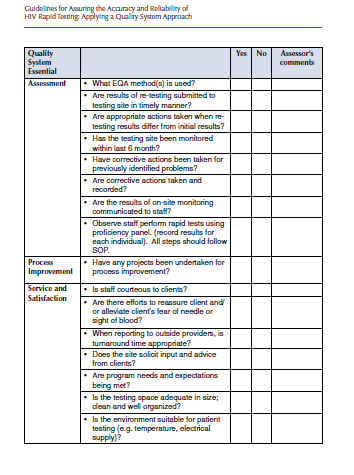


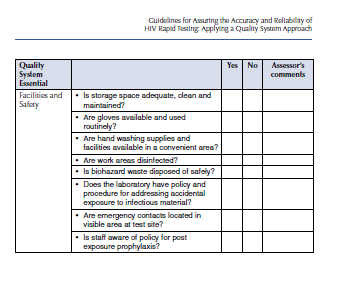

Supplement: S2 Table — (DOCX) [file pone.0183044.s002.docx]
